# Comparison between Chinese Herbal Medicines and Conventional Therapy in the Treatment of Severe Hand, Foot, and Mouth Disease: A Randomized Controlled Trial

**DOI:** 10.1155/2014/140764

**Published:** 2014-02-27

**Authors:** Xiuhui Li, Xi Zhang, Jianbo Ding, Yi Xu, Dan Wei, Yimei Tian, Wei Chen, Jihan Huang, Tao Wen, Shuangjie Li

**Affiliations:** ^1^Department of Integrated TCM and Western Medicine, Beijing Youan Hospital Affiliated with Capital Medical University, Beijing 100069, China; ^2^Department of Pediatrics, Kaifeng Children's Hospital, Kaifeng, Henan 475003, China; ^3^Department of Pediatrics, Guangzhou Medical Center for Women & Children, Guangzhou, Guangdong 510623, China; ^4^Department of Pediatrics, First Hospital Affiliated with Guangxi Medical University, Nanning, Guangxi 530021, China; ^5^Centre for Evidence-Based Chinese Medicine, Beijing University of Chinese Medicine, Beijing 100700, China; ^6^Center for Drug Clinical Research, Shanghai University of Chinese Medicine, Shanghai 201203, China; ^7^Beijing Institute of Hepatology, Beijing Youan Hospital Affiliated with Capital Medical University, Beijing 100069, China; ^8^Department of Communicable Diseases, Children's Hospital of Hunan Province, Changsha, Hunan 410007, China

## Abstract

*Background.* This study was made to evaluate the efficacy of Chinese herbal medicines, Reduning injection, and a traditional Chinese medicine (TCM) granule, in patients with severe hand, foot, and mouth disease (HFMD) by conducting a prospective, controlled, and randomized trial.* Methods.* 355 severe HFMD patients were randomly assigned to receive conventional therapy alone, Reduning injection plus conventional therapy, or TCM enema plus conventional therapy for 7–10 days. *Results.* There was no significant difference in the incidence of major complications between the groups. Median time to fever clearance was 20 hours (95% CI: 6.0–25.0) for conventional therapy recipients, 18 hours (95% CI: 4.0–24.0) for Reduning combination-treated patients, and 6 hours (95% CI: 4.0–16.0) for TCM combination-treated patients. Only the difference in time to fever clearance between TCM combination group and conventional group reached statistical significance (*P* = 0.048). Reduning combination group showed a significant reduction in sedative administration compared with conventional therapy group (*P* = 0.008). No HFMD-related death and no important adverse events were observed. *Conclusions.* Reduning injection plus conventional therapy significantly reduced the concomitant use of sedatives, which may help decrease HFMD-related neurologic complications in children. TCM effectively reduced time to fever clearance and may become a complementary therapy for relieving the symptoms of severe HFMD.

## 1. Introduction

Hand, foot, and mouth disease (HFMD) is a common infectious disease in children that is usually caused by enterovirus 71 (EV71) or coxsackievirus type A 16 (CVA16), which account for more than 70% of cases [1–4]. The clinical manifestations of HFMD typically begin with fever, sore throat, headache, myalgia, tiredness, and anorexia. One or two days later, blisters appear in the mouth, and rashes develop on the cheeks, gums, and tongue [5, 6]. In recent years, HFMD has become more common in the Asia Pacific region, especially in China [7, 8]. A report by the Ministry of Health of China in 2010 documented more than one million HFMD cases annually and more than 2,000 HFMD-associated deaths in China over the past 3 years [9].

The illness is usually mild and self-limiting, with relief of symptoms within 7–10 days, but viruses can be detected in the stool for weeks after the symptoms dissipate [5]. It is notable that, in certain cases, HFMD can progress into a severe condition with life-threatening complications, including aseptic meningitis and encephalitis, neurogenic pulmonary edema, pulmonary hemorrhage, and circulatory failure [10, 11]. There are no specific treatments for HFMD. The most common treatment is only to relieve symptoms by supportive therapy and the use of antivirals, antibiotics, and immunopotentiators [1, 5]. However, medications such as antivirals have not demonstrated convincing clinical efficacy, particularly in treating severe HFMD [1, 2, 5], and there is no vaccine currently available. Therefore, the development of more effective treatments for severe HFMD is urgently needed.

Traditional Chinese medicine (TCM), including Chinese herbal formulas and injections, has been widely used in the treatment of HFMD, as recommended by the Ministry of Health of China in 2010 [12]. Observational studies of hospitalized patients with HFMD revealed that treatment with TCM could reduce disease severity and mortality [6, 13, 14]. Among these therapies, Reduning injection, composed of three herbs (Herba *Artemisia annua, Lonicera japonica* Thunb, and *Gardenia jasminoides* Ellis), has been used clinically to reduce the fever and inflammation associated with HFMD [6, 15]. In addition, certain TCM formulas (several herbs in combination) exhibit antiviral and immunomodulating effects [6, 16] and are used by TCM practitioners to relieve the symptoms of HFMD [14]. However, no direct comparative evidence on the efficacy and safety of Chinese herbal medicines (Reduning injection or TCM formulas) in the treatment of severe HFMD has been reported. In this study, we conducted a carefully designed prospective clinical trial with a sufficient sample size to observe the efficacy and safety of both Reduning injection and TCM formulas in conjunction with conventional therapy in treating severe HFMD. The aim of our study was to assess the complementary and alternative therapeutic potential of these two Chinese herbal medicines to encourage worldwide recognition.

## 2. Methods

### 2.1. Study Design

A prospective, randomized, controlled, and multicenter trial was conducted during the HFMD epidemic between May and August 2010 at five medical sites in China. A total of 355 eligible children with severe HFMD were recruited. The study was conducted in accordance with the Declaration of Helsinki, approved by local ethics committees and institutional review boards as appropriate and registered (clinical trials registration: NCT01145664). All patients and their guardians agreed and provided signed written informed consent forms before enrollment.

### 2.2. Patient Enrollment

The diagnosis of severe HFMD was established according to the guidelines for the diagnosis and treatment of HFMD issued by the Ministry of Health of China in 2010 [12]. In addition, the following inclusion criteria were considered: ages of 1–13 years; no more than 24 hours after the occurrence of central nervous system symptoms; with any of the following symptoms: lethargy and weakness, agitation or irritability, headache, vomiting, limb weakness or acute flaccid paralysis, myoclonic jerks, ataxia, nystagmus, and oculomotor palsies.

Patients were excluded if they had neurogenic pulmonary edema or heart or respiratory failure; had chronic hepatitis, congenital heart disease, or acute or chronic nephritis; had a history of allergy to herbal medicine; had a history of mild increases in bilirubin, with intravascular hemolysis (or uric bravery former positive); had taken Chinese herbal medicines or antivirals; were using hormonal therapy; were participating in other clinical studies on HFMD; or had any condition that could have interfered with the evaluation of the study's objectives.

### 2.3. Sample Size and Randomization

The sample size calculation is based on the incidence of major complications in severe HFMD. According to the epidemic data issued by the Ministry of Health in China in 2010 [9] and the prior randomized trial [13], the incidence of major complications in severe HFMD patients receiving conventional therapy is approximately 15% and is expected to be 5% in patients receiving conventional therapy plus Chinese herbal medicines (Reduning injection or TCM).

With an error probability of 0.05 and a power of 80%, computer formula (Drug and Statistics Software, DAS) yielded a sample size of 111 subjects per treatment arm. Considering a possible 10–18% expulsion rate, the final sample size was designed to recruit 130 subjects per group, which was assumed to provide adequate power to draw reasonable inferences about enrollment, adherence, and loss to followup [13].

Participants were randomly assigned to one of three treatment groups using a web-based randomization system which enables randomization and stratification for each center. This system was provided by China Academy of Chinese Medical Sciences, which adopted the computer telephone integration technology to integrate computer, internet, and telecom [13]. After eligibility had been determined and consent had been obtained, the trial identification number, date of birth, and trial center name were entered into this randomization system. The random number list was then generated by a statistician who was not involved in data collection or analysis. The researcher immediately informed a coordinator at each center who was blinded to the participants' characteristics to assign the participants to treatment. The participants were also blinded to the treatment assignment.

The randomization was stratified according to these stratification factors: (1) geographic region (5 study centers in total) and (2) age and gender, with an aim of obtaining an equal distribution of participants. The five study centers are located in Beijing city (capital of China), Kaifeng city (Henan province), Guangzhou city (Guangdong province), Nanning city (Guangxi province), and Changsha city (Hunan province). These centers were selected to ensure broad geographic spread and representation of HFMD epidemic areas in mainland China [13, 17].

### 2.4. Drug Administration

Conventional therapy was administered to three groups of patients according to the guidelines for the diagnosis and treatment of HFMD (China, 2010) [12], including decreasing intracranial hypertension, conscious sedation, declining temperature, and intravenous glucocorticoid and immunoglobulin use. The detailed treatments included mannitol 0.5–1.0 g/kg IV administered over 30–40 minutes every 4–8 hours; glucocorticoid methylprednisolone (1-2 mg/kg/24 h); hydrocortisone (3–5 mg/kg/24 h); dexamethasone (0.2–0.5 mg/kg/24 h); intravenous immunoglobulin (IVIG) 2 g/kg over 2–5 days (recommended for patients with encephalitis plus acute flaccid paralysis and may be considered in patients with brainstem encephalitis); and others, such as paracetamol, oxygen, and transfer to the ICU if needed.

A Chinese herbal medicine-Reduning injection that consists of the three herbs, qinghao (Herba *Artemisia annua),* jinyinhua (*Lonicera japonica* Thunb), and zhizi (*Gardenia jasminoides* Ellis), is manufactured by GMP-certified Jiangsu Kangyuan Pharmaceutical, Inc., in China. The criteria for the quality of Reduning injection were in accordance with the Chinese pharmacopoeia (2005) [18]. Reduning injection was administered to patients at 0.5 mL/kg/day by intravenous infusion (in 5% dextrose) for 7–10 days.

The TCM formulas that we used in our study were composed of 12 herbs: ling yang jiao (*Saiga tatarica* Linnaeus), gou teng (*Uncaria rhynchophylla*), tianma (*Gastrodia elata*), shi gao (*Gypsum fibrosum*), huang lian (*Coptis chinensis* Franch), zhizi (*Gardenia jasminoides* Ellis), da huang (*Rheum palmatum*), ju hua (*Chrysanthemum morifolium* Ramat), yimi (*Coix lacryma-jobi*), quan xie (*Buthus martensii* Karsch), baijiang can (*Bombyx mori* Linnaeus), and muli (*Ostrea gigas* Thunb). The criteria for the quality of the herbs that we used were in accordance with the Chinese pharmacopoeia (2005) [18]. All herbs were distributed to the five medical sites from the same source. Before commencement of the trial, the herbs were tested for heavy metals, microbial contamination, and residual pesticides; all results met the safety standards in China. At each medical site, a trained technician prepared the enema according to a standardized procedure. TCM administration was given as an enema by rectal administration.

We followed up the patients in the three groups every day in the treatment period and for 15 days in the posttreatment period.

### 2.5. Assessment of Clinical Outcomes

During hospitalization, the presence and severity of HFMD symptoms and drug-associated side effects were recorded daily. The primary clinical outcome was the incidence of one or more complications, such as aseptic meningitis, brainstem encephalitis, encephalitis, purulent meningitis, encephalomyelitis, acute flaccid paralysis, autonomic nervous system dysregulation, pulmonary edema/hemorrhage, respiratory failure, circulatory failure, cardiorespiratory failure, or any other serious adverse events. The secondary outcomes included the time to fever clearance (defined as the time until the first drop in body temperature ≤37°C that remained ≤37.0°C for at least 24 hours after the first dose of an intervention was given), the time to rash subsidence (defined as the time from the onset of a typical skin rash or oral ulcer to the time of disappearance), HFMD-related mortality, the combined utilization of intracranial pressure-lowering drugs and sedatives, and intervention-related side effects.

### 2.6. Statistical Analysis

All continuous variables were expressed as the mean ± SD and analyzed between groups by Student's *t*-test. Categorical variables were expressed as percentages and analyzed by the chi-squared test. The time to fever clearance and to rash/lesion subsidence was expressed as medians and analyzed by the log-rank test. All tests were in two-tailed, and *P* values less than 0.05 were considered statistically significant. All statistical procedures were performed with SAS software (version 9.2; SAS Institute, Inc., Cary, NC, USA).

## 3. Results

### 3.1. Participants' Characteristics

**Figure 1:**
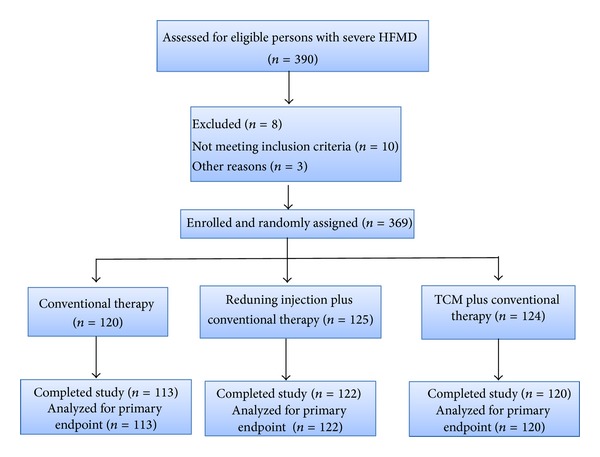
Study flow diagram.

We recruited 390 participants from five medical sites in five provinces in China. Of the 390 participants, 8 participants were excluded because their informed consents were not obtained, 10 participants were excluded because they did not meet inclusion criteria, and 3 participants were excluded because they failed the laboratory test. In total, 369 participants were actually enrolled and randomly assigned to the three groups (Figure 1).

In the conventional therapy group, 7 participants were excluded because they were found to take other herb medicines during hospitalization. In the Reduning plus conventional therapy group, 3 participants were excluded because they did not take Reduning injection. In the TCM plus conventional therapy group, 4 participants were excluded because they did not take TCM. Therefore, there were totally 355 participants who completed the trial and were included in a full analysis set (FAS). There were no loss of follow-up cases.

The baseline demographic characteristics, clinical features, and laboratory variables were similar among the three groups (Table 1).

### 3.2. Clinical Outcomes

The study showed the effects of the interventions of Chinese herbal medicines on alleviating illness, as demonstrated by fewer complications, the reduced time to fever clearance, and reduced utilization of intracranial pressure-lowering drugs and sedatives (Table 2). Major complications occurred less often after Reduning injection or TCM administration. Among 355 patients, 11 complications (3.1%) were recorded. There were five cases of major complications occurring in the conventional therapy group (4.4%), three cases occurring in the Reduning combination therapy group (2.5%), and three cases occurring in the TCM combination group (2.5%). The differences between groups did not reach statistical significance (4.4% versus 2.5% or 2.5%, *P* = 0.50). Compared with conventional therapy, fewer patients who received Reduning injection or TCM developed neurogenic edema (1.8% versus 1.6% or 0.8%) or bacterial infection (1.8% versus 0% or 0.8%), but there was no statistical difference between groups.

**Figure 2:**
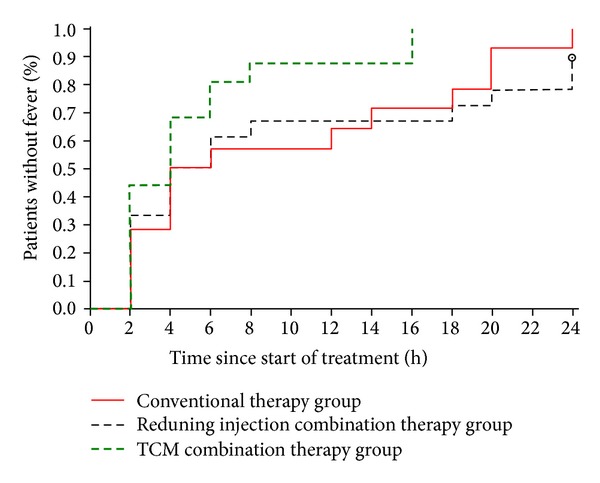
Kaplan-Meier curves of time from the start of treatment to the recording of a temperature ≤37.0°C for the subsequent 24 hours for three groups.

The median time to fever clearance was reduced in both the Reduning injection combination therapy group (18 hours, 95% CI: 4.0–24.0) and the TCM combination therapy group (6 hours, 95% CI: 4.0–16.0) compared with the conventional therapy group (20 hours, 95% CI: 6.0–25.0). However, only the difference between TCM combination therapy and conventional therapy alone reached borderline statistical significance (*P* = 0.048) (Figure 2).

The time to typical skin or oral rash/lesion subsidence was 7 days (95% CI: 6.0-7.0) in the conventional therapy group, 6 days (95% CI: 6.0-7.0) in the Reduning injection combination group, and 7 days (95% CI: 6.0-7.0) in the TCM combination group. The difference was not statistically significant (*P* = 0.667).

Of note, the utilization of intracranial pressure-lowering drugs and sedatives was observed to decrease in the two intervention groups involving combination therapy. The frequency of administration of intracranial pressure-lowering drugs was 68% in the Reduning injection combination group and 70.8% in the TCM group, as opposed to 77% in the conventional therapy group, although no statistical difference was attained (*P* = 0.30). Moreover, the frequency of sedative administration was 11.5% in the Reduning combination group and 18.3% in the TCM combination group, as opposed to 24.8% in the conventional therapy group. A statistical difference was obtained between the Reduning group and the conventional group (*P* = 0.008), suggesting that Reduning injection reduced the need for sedative administration in children with severe HFMD.

There were no HFMD-caused deaths in any of the three groups. Therefore, the mortality rates could not be determined and compared. In addition, no differences in any symptoms, including cough, sore throat, headache, and fatigue, were observed among the three groups after treatment.

### 3.3. Safety

Reduning injection and TCM enema were well tolerated. During the course of treatment, there were no drug-related side effects or serious adverse events observed in the three groups.

## 4. Discussion

Several clinical trials have assessed the efficacy and safety of Chinese herbal medicines including formulas (several herbs in combination) and injections in treating HFMD [1, 6, 15]. However, there are certain limitations in these studies. First, most trials were restricted to a small sample size, which yielded bias and did not reflect the general population of patients [6]. Second, the findings of these trials were mostly reported in Chinese journals and not translated into English and thus cannot be well recognized around the world. Third, although most trials demonstrated the encouraging efficacy of herbal medicines in contrast to conventional therapy in common HFMD patients, severe HFMD patients were not proportionally investigated [6]. To the best of our knowledge, this is one of the first registered prospective, randomized, and controlled trials to evaluate the efficacy and safety of herbal medicines, such as Reduning injection and TCM formulas, in severe HFMD treatment. We found that the Chinese herbal medicines in the form of Reduning injection and TCM enema show potential as a complementary therapy for severe HFMD. In conjunction with conventional therapy, Reduning injection significantly reduced the need for sedative administration, whereas the TCM formulas shortened the time to fever clearance in the treatment of severe HFMD. However, some clinical improvements, such as the decreased incidence of major complications, were not significantly different, most likely due to the very small number of cases with major complications. The study also showed that the disappearance of the typical rash/lesions was faster with Reduning injection or TCM enema combined intervention, but the difference was not statistically significant. Overall, regarding clinical implications, we may suggest Reduning injection or a TCM formula as a complementary rather than alternative medication that may be introduced to resolve several particular problems in certain clinical situations. For example, Reduning injection may be recommended when patients with severe HFMD start to show neurological symptoms.

Reduning injection, one of the famous traditional Chinese preparations, consists of three herbs, Herba *Artemisia annua*, *Lonicera japonica* Thunb, and *Gardenia jasminoides* Ellis [15]. The mechanism of Reduning injection in the treatment of HFMD is yet to be elucidated. It has been reported that *Artemisia annua* has numerous biological functions such as antimicrobial and antiparasitic properties [16, 19]. *Lonicera japonica* Thunb and *Gardenia jasminoides* are known for their abilities to remove heat and toxic materials and to relieve inflammation. These herbs are widely used as important components in TCM formulas in clinical settings [20, 21]. It is speculated that the synergy of the three individual herbs is responsible for the therapeutic efficacy of Reduning injection. Indeed, Reduning injection has been used in the treatment of common HFMD and showed improvement in relieving the symptoms such as body temperature [15]. However, in the treatment of severe HFMD cases in this clinical trial, we did not observe a similarly significant improvement in the time to fever resolution or the time to rash/lesions disappearance. However, we did find evidence of differences in the reduction in the concomitant administration of sedatives. It is well known that severe HFMD is usually accompanied by neurological disorders/complications that may cause death if left untreated [5, 10]. Administration of a sedative is very commonly performed to relieve symptoms among children with severe HFMD [12], but it may cause side effects such as dizziness, lethargy, and disorientation, and it will affect children's growth and development in the long run [22]. Therefore, the reduction in sedative administration by Reduning injection will be meaningful and beneficial to the children, which should be highlighted in clinical practice.

In our study, TCM showed a degree of efficacy in accelerating the median time to fever clearance, which suggested that TCM can serve as a complementary therapy to conventional therapy, as antipyretic treatment is indeed one of the major therapies for HFMD [1, 12]. The TCM formula that we used in this study was recommended by the Ministry of Health in China in 2010 [12]. However, the bioactive compounds that are responsible for the formula's therapeutic effects are largely unclear. It was reported that *Coptis chinensis* Franch has similar anti-inflammatory and detoxication effects as *Gardenia jasminoides* Ellis, both of which are widely used in other formulas to treat H1N1 influenza [17, 21]. In addition, several other herbs, including *Gastrodia elata* and *Rheum palmatum,* have inhibitory effects on influenza virus A by interfering with virus proliferation and protecting cells from being infected with the virus [21, 23]. Although it is unclear whether these herbs have a similar effect on HFMD-related viruses, the identified immunomodulatory effects of these herbs may help to shorten the time to fever clearance in HFMD. Generally, all of the herbs are assumed to play a synergistic role in producing maximum therapeutic efficacy. More rigorous studies are needed to accurately clarify the mechanisms of TCM in treating HFMD.

Generally, TCM is prepared as decoctions to administer to patients [6]. However, for children with HFMD it is very difficult to conduct oral administration because these patients usually have oral ulcers or blisters and are also resistant to decoctions due to the preparations' bitterness. Therefore, we prepared TCM as an enema administered to children via the rectum. TCM enema is easier to prepare and administer and thus may become a preferred method in clinical settings.

There are certain limitations in this study. First, this study was not a double-blind, placebo-controlled clinical trial. Reduning was given as an injection, whereas TCM enema was given by rectal administration. We could not find appropriate placebos for these Chinese herbal medicines before the trial began. As this trial focused on children with severe HFMD, more caution should be taken to decrease the possible risks to a minimum, and a placebo-controlled design might not be applicable. Besides, assessment of clinical outcomes such as measuring the incidence of complications, body temperature, and the combined utilization of some medicines were objective findings, and the nurses responsible for such measurement were unaware of study group assignment, which thus ensured the observed effect was objective and real. Second, no adverse events were reported in our study, most likely because such adverse events as vomiting, diarrhea, nausea, and headache were common in all three groups. It is difficult to determine whether these events were HFMD-related or intervention-related events. Third, we only collected outcome measures for 15 days after enrollment for all patients. Without a long-term followup, it is hard to conclude whether Reduning injection or TCM for severe HFMD treatment is safe in the long term.

## 5. Conclusions

In summary, our study provides evidence that both Reduning injection and TCM formulas can be safely used in the treatment of severe HFMD. Combination therapy will help to shorten the time to fever clearance and to reduce the concomitant use of sedatives, thus accelerating the resolution of symptoms, and can serve as a complementary medicine.

## Figures and Tables

**Table 1:** Characteristics of participants with severe HFMD.

Characteristic	Conventional therapy group (*n* = 113)	Reduning injection combination therapy group (*n* = 122)	TCM formulas combination therapy group (*n* = 120)
Male, *n* (%)	73 (64.6)	83 (68.0)	79 (65.8)
Mean age ± SD, y	1.84 ± 0.96	1.98 ± 1.33	1.91 ± 1.15
Temperature, *n* (%)			
37.5–38.0°C	38 (33.6)	42 (34.4)	41 (34.2)
38.1–39.0°C	60 (53.1)	58 (47.5)	62 (51.7)
>39.0°C	15 (13.3)	22 (18.0)	17 (14.2)
Symptom, *n* (%)			
Cough	9 (8.0)	9 (7.4)	13 (10.9)
Sleepiness	29 (26.1)	25 (20.8)	27 (22.7)
Headache	3 (2.7)	6 (5.0)	4 (3.4)
Irritability	11 (9.8)	12 (10.0)	10 (8.5)
Fatigue	66 (58.9)	69 (58.0)	73 (60.8)
Skin or oral mucosal lesions	76 (67.2)	70 (57.4)	79 (65.8)
Height (SD), cm	87.43 (13.48)	88.53 (14.34)	87.14 (11.47)
Body weight (SD), kg	12.68 (2.94)	12.93 (3.75)	12.61 (3.07)

**Table 2:** Clinical outcomes for participants with severe HFMD.

Variable	Conventional therapy group (*n* = 113)	Reduning injection combination therapy group (*n* = 122)	TCM formulas combination therapy group (*n* = 120)	*P* value of three groups' comparison	*P* value of two groups' comparison
Primary outcome					
Major complications, *n* (%)	5 (4.4)	3 (2.5)	3 (2.5)	0.890	
Neurogenic pulmonary edema	0 (0)	1 (0.8)	1 (0.8)	0.626	
Cerebral edema	2 (1.8)	2 (1.6)	1 (0.8)	0.803	
Bacterial infection	2 (1.8)	0 (0)	1 (0.8)	0.335	
Circulatory failure	1 (0.9)	0 (0)	0 (0)	0.343	
Secondary outcome					
Time to fever clearance, h (95% CI)	20 (6.0–25.0)	18 (4.0–24.0)	6 (4.0–16.0)	0.0493	0.048 (TCM group relative to conventional group)
Time to rash subsidence, days (95% CI)	7 (6.0-7.0)	6 (6.0-7.0)	7 (6.0-7.0)	0.667	
HFMD-related mortality	0	0	0	1.000	
Combined utilization of intracranial pressure-lowering drugs	77%	60.8%	68%	0.30	
Combined utilization of sedatives, %	24.8%	11.5%	18.3	0.012	0.008 (Reduning group relative to conventional group)
Adverse event, *n* (%)	0	0	0	1.000	
